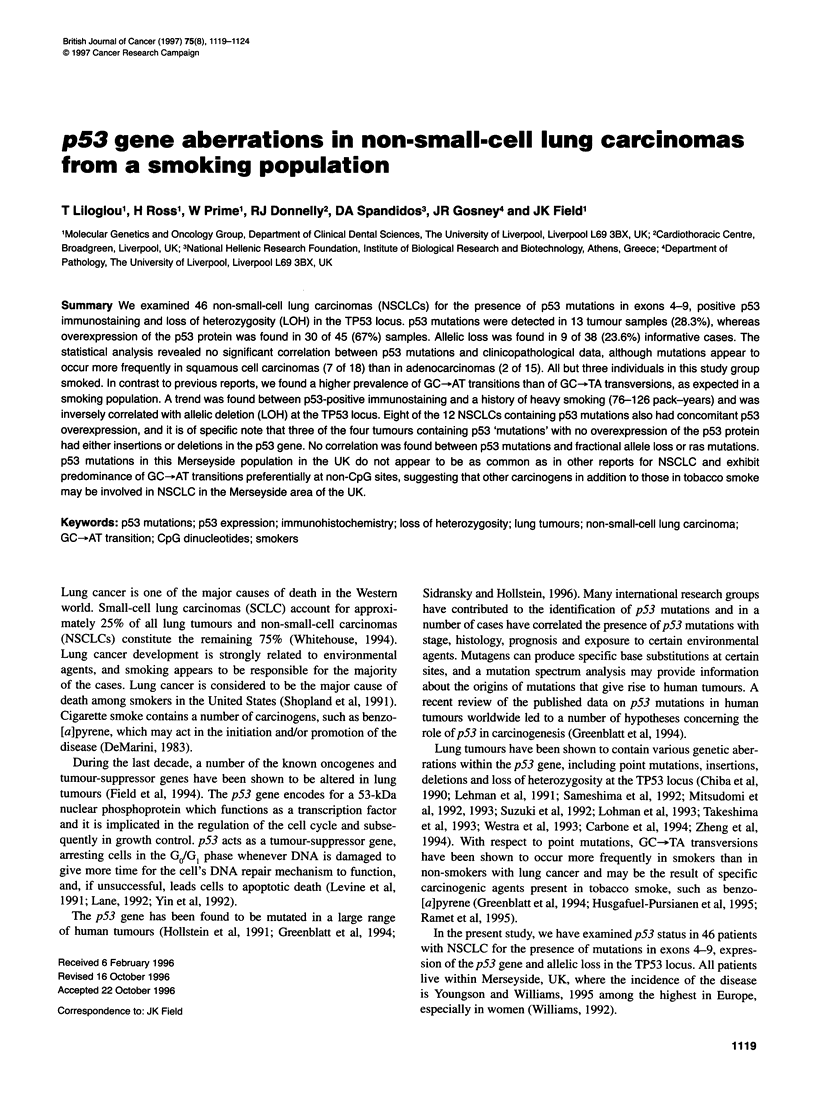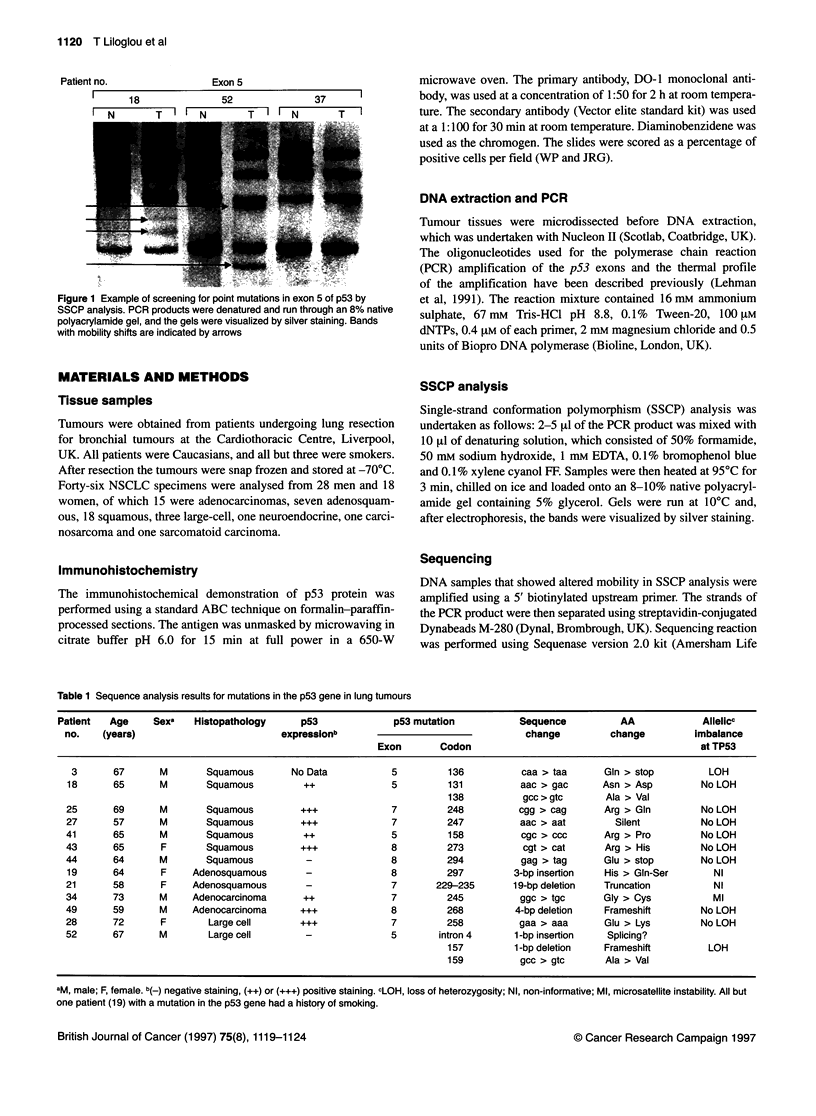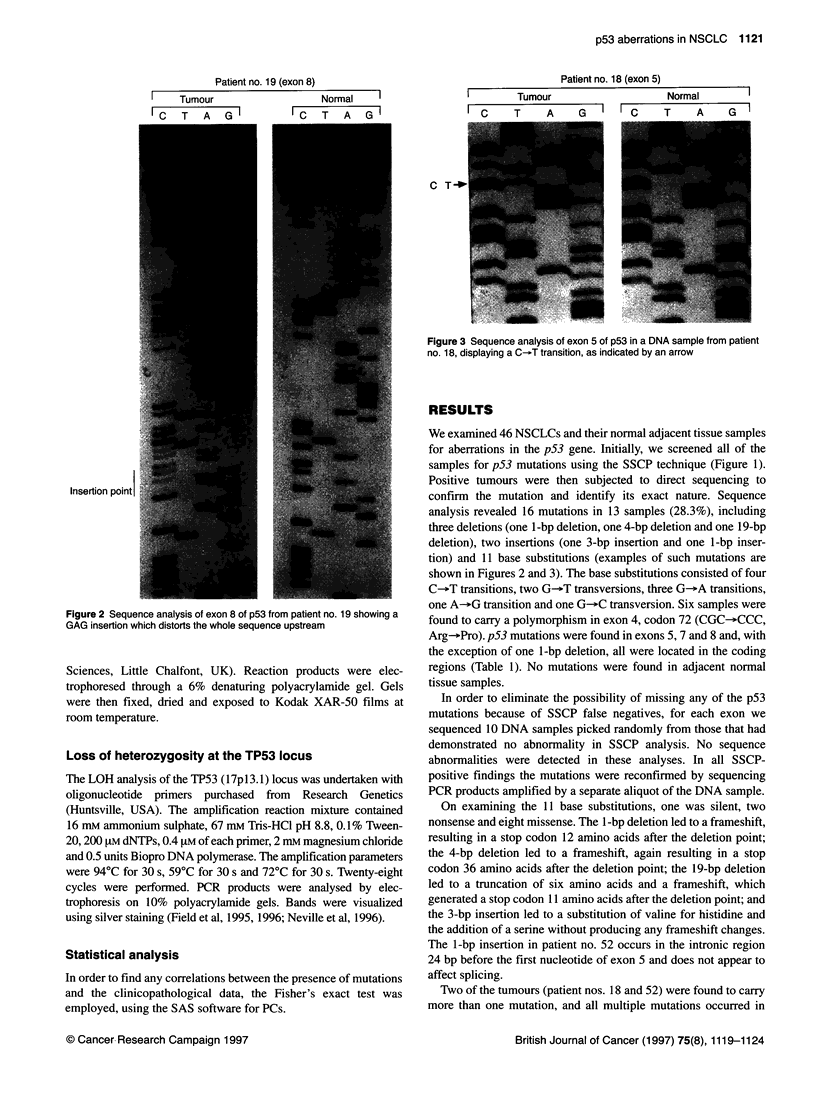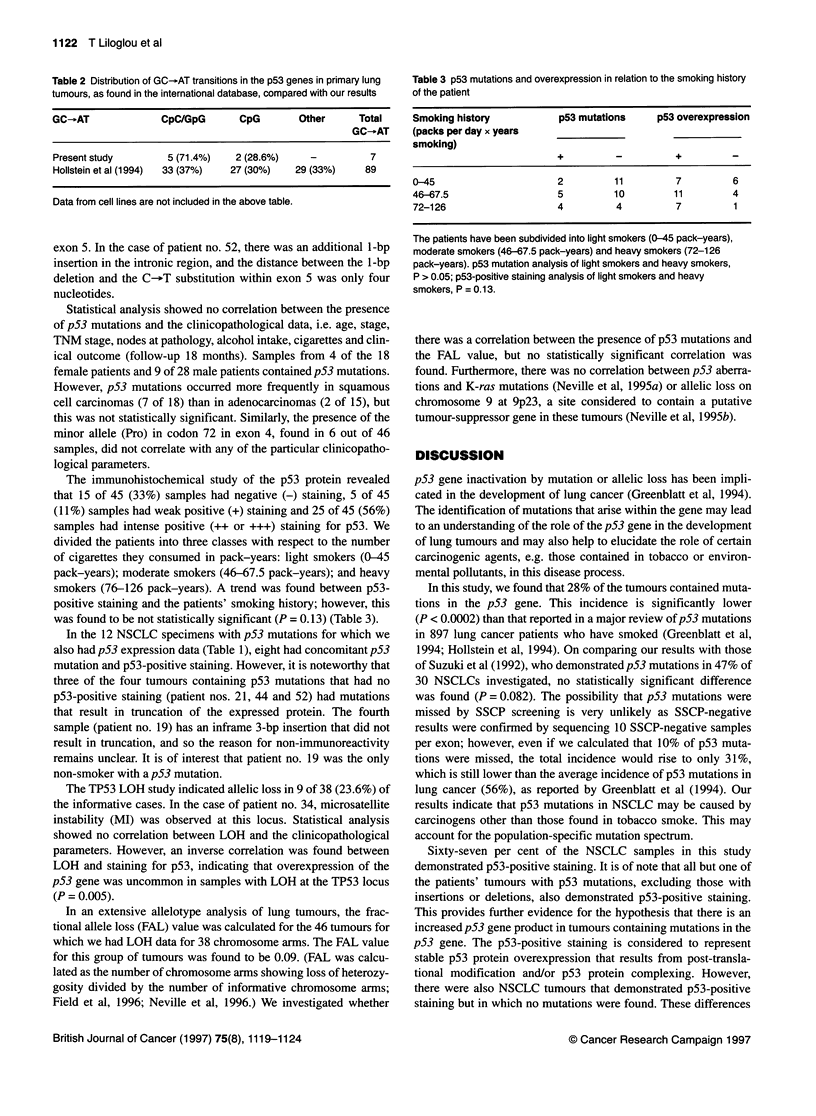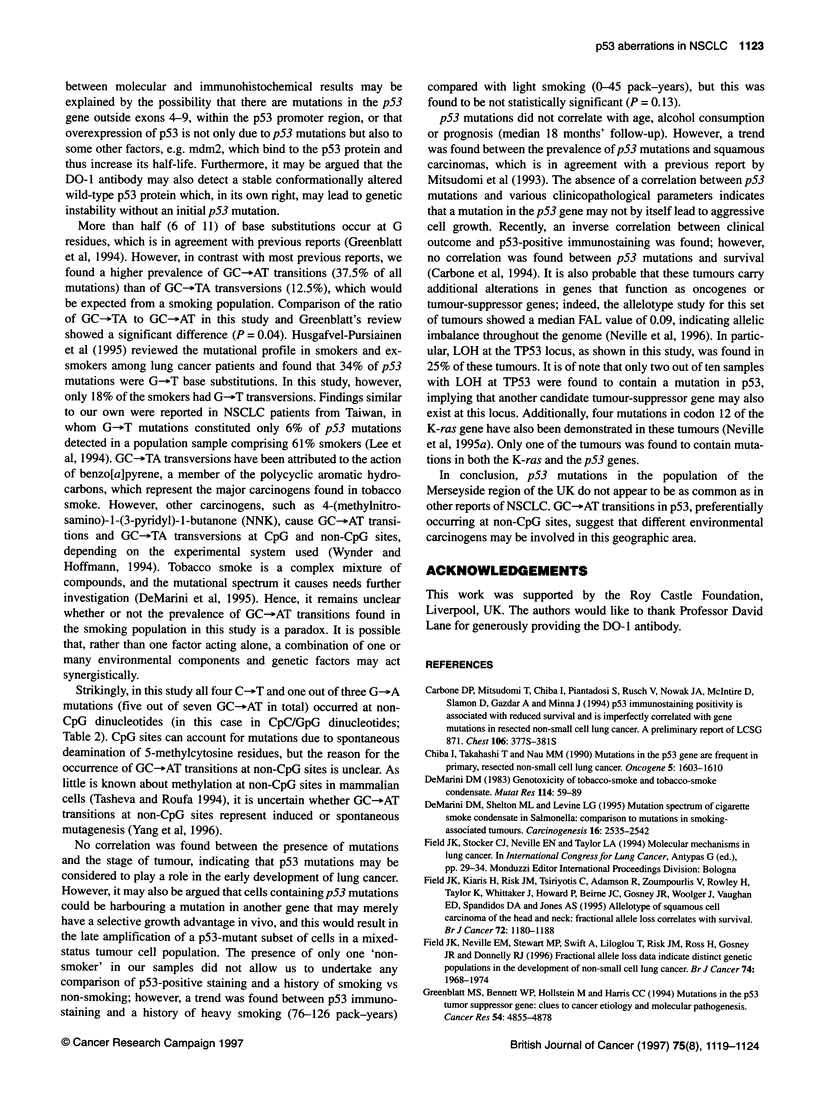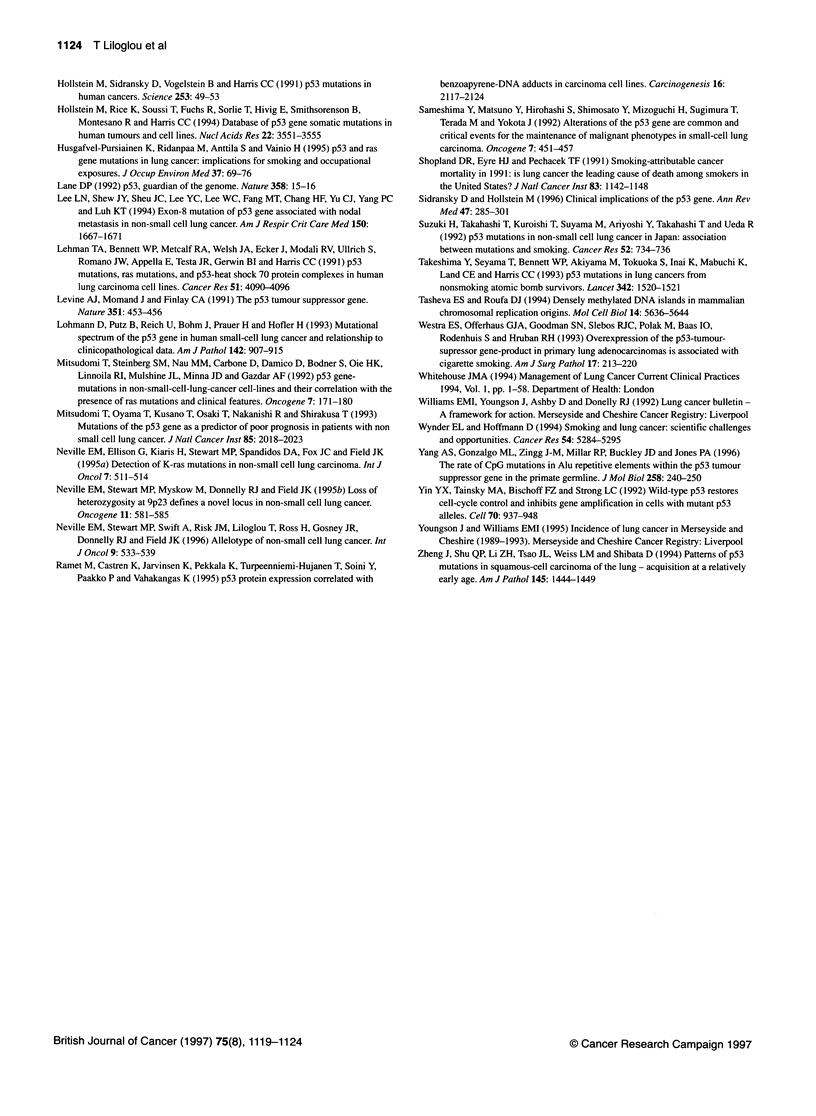# p53 gene aberrations in non-small-cell lung carcinomas from a smoking population.

**DOI:** 10.1038/bjc.1997.193

**Published:** 1997

**Authors:** T. Liloglou, H. Ross, W. Prime, R. J. Donnelly, D. A. Spandidos, J. R. Gosney, J. K. Field

**Affiliations:** Department of Clinical Dental Sciences, The University of Liverpool, UK.

## Abstract

**Images:**


British Journal of Cancer (1997) 75(8), 1119–1124
© 1997 Cancer Research Campaign

### *p53* gene aberrations in non-small-cell lung carcinomas from a smoking population

**T Liloglou[1], H Ross[1], W Prime[1], RJ Donnelly[2], DA Spandidos[3], JR Gosney[4] and JK Field[1]**

[1]Molecular Genetics and Oncology Group, Department of Clinical Dental Sciences, The University of Liverpool, Liverpool L69 3BX, UK; [2]Cardiothoracic Centre, Broadgreen, Liverpool, UK; [3]National Hellenic Research Foundation, Institute of Biological Research and Biotechnology, Athens, Greece; [4]Department of Pathology, The University of Liverpool, Liverpool L69 3BX, UK

**Summary** We examined 46 non-small-cell lung carcinomas (NSCLCs) for the presence of p53 mutations in exons 4–9, positive p53 immunostaining and loss of heterozygosity (LOH) in the TP53 locus. p53 mutations were detected in 13 tumour samples (28.3%), whereas overexpression of the p53 protein was found in 30 of 45 (67%) samples. Allelic loss was found in 9 of 38 (23.6%) informative cases. The statistical analysis revealed no significant correlation between p53 mutations and clinicopathological data, although mutations appear to occur more frequently in squamous cell carcinomas (7 of 18) than in adenocarcinomas (2 of 15). All but three individuals in this study group smoked. In contrast to previous reports, we found a higher prevalence of GC→AT transitions than of GC→TA transversions, as expected in a smoking population. A trend was found between p53-positive immunostaining and a history of heavy smoking (76–126 pack–years) and was inversely correlated with allelic deletion (LOH) at the TP53 locus. Eight of the 12 NSCLCs containing p53 mutations also had concomitant p53 overexpression, and it is of specific note that three of the four tumours containing p53 'mutations' with no overexpression of the p53 protein had either insertions or deletions in the p53 gene. No correlation was found between p53 mutations and fractional allele loss or ras mutations. p53 mutations in this Merseyside population in the UK do not appear to be as common as in other reports for NSCLC and exhibit predominance of GC→AT transitions preferentially at non-CpG sites, suggesting that other carcinogens in addition to those in tobacco smoke may be involved in NSCLC in the Merseyside area of the UK.

**Keywords:** p53 mutations; p53 expression; immunohistochemistry; loss of heterozygosity; lung tumours; non-small-cell lung carcinoma; GC→AT transition; CpG dinucleotides; smokers

Lung cancer is one of the major causes of death in the Western world. Small-cell lung carcinomas (SCLC) account for approximately 25% of all lung tumours and non-small-cell carcinomas (NSCLCs) constitute the remaining 75% (Whitehouse, 1994). Lung cancer development is strongly related to environmental agents, and smoking appears to be responsible for the majority of the cases. Lung cancer is considered to be the major cause of death among smokers in the United States (Shopland et al, 1991). Cigarette smoke contains a number of carcinogens, such as benzo-[*a*]pyrene, which may act in the initiation and/or promotion of the disease (DeMarini, 1983).

During the last decade, a number of the known oncogenes and tumour-suppressor genes have been shown to be altered in lung tumours (Field et al, 1994). The *p53* gene encodes for a 53-kDa nuclear phosphoprotein which functions as a transcription factor and it is implicated in the regulation of the cell cycle and subsequently in growth control. *p53* acts as a tumour-suppressor gene, arresting cells in the $G_0/G_1$ phase whenever DNA is damaged to give more time for the cell's DNA repair mechanism to function, and, if unsuccessful, leads cells to apoptotic death (Levine et al, 1991; Lane, 1992; Yin et al, 1992).

The *p53* gene has been found to be mutated in a large range of human tumours (Hollstein et al, 1991; Greenblatt et al, 1994; Sidransky and Hollstein, 1996). Many international research groups have contributed to the identification of *p53* mutations and in a number of cases have correlated the presence of *p53* mutations with stage, histology, prognosis and exposure to certain environmental agents. Mutagens can produce specific base substitutions at certain sites, and a mutation spectrum analysis may provide information about the origins of mutations that give rise to human tumours. A recent review of the published data on *p53* mutations in human tumours worldwide led to a number of hypotheses concerning the role of *p53* in carcinogenesis (Greenblatt et al, 1994).

Received 6 February 1996
Revised 16 October 1996
Accepted 22 October 1996

Correspondence to: JK Field

Lung tumours have been shown to contain various genetic aberrations within the *p53* gene, including point mutations, insertions, deletions and loss of heterozygosity at the TP53 locus (Chiba et al, 1990; Lehman et al, 1991; Sameshima et al, 1992; Mitsudomi et al, 1992, 1993; Suzuki et al, 1992; Lohman et al, 1993; Takeshima et al, 1993; Westra et al, 1993; Carbone et al, 1994; Zheng et al, 1994). With respect to point mutations, GC→TA transversions have been shown to occur more frequently in smokers than in non-smokers with lung cancer and may be the result of specific carcinogenic agents present in tobacco smoke, such as benzo-[*a*]pyrene (Greenblatt et al, 1994; Husgafuel-Pursianen et al, 1995; Ramet et al, 1995).

In the present study, we have examined *p53* status in 46 patients with NSCLC for the presence of mutations in exons 4–9, expression of the *p53* gene and allelic loss in the TP53 locus. All patients live within Merseyside, UK, where the incidence of the disease is Youngson and Williams, 1995 among the highest in Europe, especially in women (Williams, 1992).

**Figure 1** Example of screening for point mutations in exon 5 of p53 by SSCP analysis. PCR products were denatured and run through an 8% native polyacrylamide gel, and the gels were visualized by silver staining. Bands with mobility shifts are indicated by arrows

### MATERIALS AND METHODS

### Tissue samples

Tumours were obtained from patients undergoing lung resection for bronchial tumours at the Cardiothoracic Centre, Liverpool, UK. All patients were Caucasians, and all but three were smokers. After resection the tumours were snap frozen and stored at –70°C. Forty-six NSCLC specimens were analysed from 28 men and 18 women, of which 15 were adenocarcinomas, seven adenosquamous, 18 squamous, three large-cell, one neuroendocrine, one carcinosarcoma and one sarcomatoid carcinoma.

### Immunohistochemistry

The immunohistochemical demonstration of p53 protein was performed using a standard ABC technique on formalin–paraffin-processed sections. The antigen was unmasked by microwaving in citrate buffer pH 6.0 for 15 min at full power in a 650-W microwave oven. The primary antibody, DO-1 monoclonal antibody, was used at a concentration of 1:50 for 2 h at room temperature. The secondary antibody (Vector elite standard kit) was used at a 1:100 for 30 min at room temperature. Diaminobenzidene was used as the chromogen. The slides were scored as a percentage of positive cells per field (WP and JRG).

### DNA extraction and PCR

Tumour tissues were microdissected before DNA extraction, which was undertaken with Nucleon II (Scotlab, Coatbridge, UK). The oligonucleotides used for the polymerase chain reaction (PCR) amplification of the *p53* exons and the thermal profile of the amplification have been described previously (Lehman et al, 1991). The reaction mixture contained 16 mM ammonium sulphate, 67 mM Tris-HCl pH 8.8, 0.1% Tween-20, 100 μM dNTPs, 0.4 μM of each primer, 2 mM magnesium chloride and 0.5 units of Biopro DNA polymerase (Bioline, London, UK).

### SSCP analysis

Single-strand conformation polymorphism (SSCP) analysis was undertaken as follows: 2–5 μl of the PCR product was mixed with 10 μl of denaturing solution, which consisted of 50% formamide, 50 mM sodium hydroxide, 1 mM EDTA, 0.1% bromophenol blue and 0.1% xylene cyanol FF. Samples were then heated at 95°C for 3 min, chilled on ice and loaded onto an 8–10% native polyacrylamide gel containing 5% glycerol. Gels were run at 10°C and, after electrophoresis, the bands were visualized by silver staining.

### Sequencing

DNA samples that showed altered mobility in SSCP analysis were amplified using a 5′ biotinylated upstream primer. The strands of the PCR product were then separated using streptavidin-conjugated Dynabeads M-280 (Dynal, Brombrough, UK). Sequencing reaction was performed using Sequenase version 2.0 kit (Amersham Life

**Table 1** Sequence analysis results for mutations in the p53 gene in lung tumours

| Patient no. | Age (years) | Sex[a] | Histopathology | p53 expression[b] | p53 mutation | | Sequence change | AA change | Allelic[c] imbalance at TP53 |
|---|---|---|---|---|---|---|---|---|---|
| | | | | | Exon | Codon | | | |
| 3 | 67 | M | Squamous | No Data | 5 | 136 | caa > taa | Gln > stop | LOH |
| 18 | 65 | M | Squamous | ++ | 5 | 131 | aac > gac | Asn > Asp | No LOH |
| | | | | | | 138 | gcc > gtc | Ala > Val | |
| 25 | 69 | M | Squamous | +++ | 7 | 248 | cgg > cag | Arg > Gln | No LOH |
| 27 | 57 | M | Squamous | +++ | 7 | 247 | aac > aat | Silent | No LOH |
| 41 | 65 | M | Squamous | ++ | 5 | 158 | cgc > ccc | Arg > Pro | No LOH |
| 43 | 65 | F | Squamous | +++ | 8 | 273 | cgt > cat | Arg > His | No LOH |
| 44 | 64 | M | Squamous | – | 8 | 294 | gag > tag | Glu > stop | No LOH |
| 19 | 64 | F | Adenosquamous | – | 8 | 297 | 3-bp insertion | His > Gln-Ser | NI |
| 21 | 58 | F | Adenosquamous | – | 7 | 229–235 | 19-bp deletion | Truncation | NI |
| 34 | 73 | M | Adenocarcinoma | ++ | 7 | 245 | ggc > tgc | Gly > Cys | MI |
| 49 | 59 | M | Adenocarcinoma | +++ | 8 | 268 | 4-bp deletion | Frameshift | No LOH |
| 28 | 72 | F | Large cell | +++ | 7 | 258 | gaa > aaa | Glu > Lys | No LOH |
| 52 | 67 | M | Large cell | – | 5 | intron 4 | 1-bp insertion | Splicing? | |
| | | | | | | 157 | 1-bp deletion | Frameshift | LOH |
| | | | | | | 159 | gcc > gtc | Ala > Val | |

[a]M, male; F, female. [b](–) negative staining, (++) or (+++) positive staining. [c]LOH, loss of heterozygosity; NI, non-informative; MI, microsatellite instability. All but one patient (19) with a mutation in the p53 gene had a history of smoking.

© Cancer Research Campaign 1997

Figure 2 Sequence analysis of exon 8 of p53 from patient no. 19 showing a GAG insertion which distorts the whole sequence upstream

Figure 3 Sequence analysis of exon 5 of p53 in a DNA sample from patient no. 18, displaying a C→T transition, as indicated by an arrow

Sciences, Little Chalfont, UK). Reaction products were electrophoresed through a 6% denaturing polyacrylamide gel. Gels were then fixed, dried and exposed to Kodak XAR-50 films at room temperature.

### Loss of heterozygosity at the TP53 locus

The LOH analysis of the TP53 (17p13.1) locus was undertaken with oligonucleotide primers purchased from Research Genetics (Huntsville, USA). The amplification reaction mixture contained 16 mM ammonium sulphate, 67 mM Tris-HCl pH 8.8, 0.1% Tween-20, 200 μM dNTPs, 0.4 μM of each primer, 2 mM magnesium chloride and 0.5 units Biopro DNA polymerase. The amplification parameters were 94°C for 30 s, 59°C for 30 s and 72°C for 30 s. Twenty-eight cycles were performed. PCR products were analysed by electrophoresis on 10% polyacrylamide gels. Bands were visualized using silver staining (Field et al, 1995, 1996; Neville et al, 1996).

### Statistical analysis

In order to find any correlations between the presence of mutations and the clinicopathological data, the Fisher's exact test was employed, using the SAS software for PCs.

### RESULTS

We examined 46 NSCLCs and their normal adjacent tissue samples for aberrations in the *p53* gene. Initially, we screened all of the samples for *p53* mutations using the SSCP technique (Figure 1). Positive tumours were then subjected to direct sequencing to confirm the mutation and identify its exact nature. Sequence analysis revealed 16 mutations in 13 samples (28.3%), including three deletions (one 1-bp deletion, one 4-bp deletion and one 19-bp deletion), two insertions (one 3-bp insertion and one 1-bp insertion) and 11 base substitutions (examples of such mutations are shown in Figures 2 and 3). The base substitutions consisted of four C→T transitions, two G→T transversions, three G→A transitions, one A→G transition and one G→C transversion. Six samples were found to carry a polymorphism in exon 4, codon 72 (CGC→CCC, Arg→Pro). *p53* mutations were found in exons 5, 7 and 8 and, with the exception of one 1-bp deletion, all were located in the coding regions (Table 1). No mutations were found in adjacent normal tissue samples.

In order to eliminate the possibility of missing any of the p53 mutations because of SSCP false negatives, for each exon we sequenced 10 DNA samples picked randomly from those that had demonstrated no abnormality in SSCP analysis. No sequence abnormalities were detected in these analyses. In all SSCP-positive findings the mutations were reconfirmed by sequencing PCR products amplified by a separate aliquot of the DNA sample.

On examining the 11 base substitutions, one was silent, two nonsense and eight missense. The 1-bp deletion led to a frameshift, resulting in a stop codon 12 amino acids after the deletion point; the 4-bp deletion led to a frameshift, again resulting in a stop codon 36 amino acids after the deletion point; the 19-bp deletion led to a truncation of six amino acids and a frameshift, which generated a stop codon 11 amino acids after the deletion point; and the 3-bp insertion led to a substitution of valine for histidine and the addition of a serine without producing any frameshift changes. The 1-bp insertion in patient no. 52 occurs in the intronic region 24 bp before the first nucleotide of exon 5 and does not appear to affect splicing.

Two of the tumours (patient nos. 18 and 52) were found to carry more than one mutation, and all multiple mutations occurred in

© Cancer Research Campaign 1997 

**Table 2** Distribution of GC→AT transitions in the p53 genes in primary lung tumours, as found in the international database, compared with our results

| GC→AT | CpC/GpG | CpG | Other | Total GC→AT |
|---|---|---|---|---|
| Present study | 5 (71.4%) | 2 (28.6%) | – | 7 |
| Hollstein et al (1994) | 33 (37%) | 27 (30%) | 29 (33%) | 89 |

Data from cell lines are not included in the above table.

**Table 3** p53 mutations and overexpression in relation to the smoking history of the patient

| Smoking history (packs per day × years smoking) | p53 mutations | | p53 overexpression | |
|---|---|---|---|---|
| | + | – | + | – |
| 0–45 | 2 | 11 | 7 | 6 |
| 46–67.5 | 5 | 10 | 11 | 4 |
| 72–126 | 4 | 4 | 7 | 1 |

The patients have been subdivided into light smokers (0–45 pack–years), moderate smokers (46–67.5 pack–years) and heavy smokers (72–126 pack–years). p53 mutation analysis of light smokers and heavy smokers, $P > 0.05$; p53-positive staining analysis of light smokers and heavy smokers, $P = 0.13$.

exon 5. In the case of patient no. 52, there was an additional 1-bp insertion in the intronic region, and the distance between the 1-bp deletion and the C→T substitution within exon 5 was only four nucleotides.

Statistical analysis showed no correlation between the presence of *p53* mutations and the clinicopathological data, i.e. age, stage, TNM stage, nodes at pathology, alcohol intake, cigarettes and clinical outcome (follow-up 18 months). Samples from 4 of the 18 female patients and 9 of 28 male patients contained *p53* mutations. However, *p53* mutations occurred more frequently in squamous cell carcinomas (7 of 18) than in adenocarcinomas (2 of 15), but this was not statistically significant. Similarly, the presence of the minor allele (Pro) in codon 72 in exon 4, found in 6 out of 46 samples, did not correlate with any of the particular clinicopathological parameters.

The immunohistochemical study of the p53 protein revealed that 15 of 45 (33%) samples had negative (–) staining, 5 of 45 (11%) samples had weak positive (+) staining and 25 of 45 (56%) samples had intense positive (++ or +++) staining for p53. We divided the patients into three classes with respect to the number of cigarettes they consumed in pack–years: light smokers (0–45 pack–years); moderate smokers (46–67.5 pack–years); and heavy smokers (76–126 pack–years). A trend was found between p53-positive staining and the patients' smoking history; however, this was found to be not statistically significant ($P = 0.13$) (Table 3).

In the 12 NSCLC specimens with *p53* mutations for which we also had *p53* expression data (Table 1), eight had concomitant *p53* mutation and p53-positive staining. However, it is noteworthy that three of the four tumours containing p53 mutations that had no p53-positive staining (patient nos. 21, 44 and 52) had mutations that result in truncation of the expressed protein. The fourth sample (patient no. 19) has an inframe 3-bp insertion that did not result in truncation, and so the reason for non-immunoreactivity remains unclear. It is of interest that patient no. 19 was the only non-smoker with a *p53* mutation.

The TP53 LOH study indicated allelic loss in 9 of 38 (23.6%) of the informative cases. In the case of patient no. 34, microsatellite instability (MI) was observed at this locus. Statistical analysis showed no correlation between LOH and the clinicopathological parameters. However, an inverse correlation was found between LOH and staining for p53, indicating that overexpression of the *p53* gene was uncommon in samples with LOH at the TP53 locus ($P = 0.005$).

In an extensive allelotype analysis of lung tumours, the fractional allele loss (FAL) value was calculated for the 46 tumours for which we had LOH data for 38 chromosome arms. The FAL value for this group of tumours was found to be 0.09. (FAL was calculated as the number of chromosome arms showing loss of heterozygosity divided by the number of informative chromosome arms; Field et al, 1996; Neville et al, 1996.) We investigated whether there was a correlation between the presence of p53 mutations and the FAL value, but no statistically significant correlation was found. Furthermore, there was no correlation between *p53* aberrations and K-*ras* mutations (Neville et al, 1995*a*) or allelic loss on chromosome 9 at 9p23, a site considered to contain a putative tumour-suppressor gene in these tumours (Neville et al, 1995*b*).

### DISCUSSION

*p53* gene inactivation by mutation or allelic loss has been implicated in the development of lung cancer (Greenblatt et al, 1994). The identification of mutations that arise within the gene may lead to an understanding of the role of the *p53* gene in the development of lung tumours and may also help to elucidate the role of certain carcinogenic agents, e.g. those contained in tobacco or environmental pollutants, in this disease process.

In this study, we found that 28% of the tumours contained mutations in the *p53* gene. This incidence is significantly lower ($P < 0.0002$) than that reported in a major review of *p53* mutations in 897 lung cancer patients who have smoked (Greenblatt et al, 1994; Hollstein et al, 1994). On comparing our results with those of Suzuki et al (1992), who demonstrated *p53* mutations in 47% of 30 NSCLCs investigated, no statistically significant difference was found ($P = 0.082$). The possibility that *p53* mutations were missed by SSCP screening is very unlikely as SSCP-negative results were confirmed by sequencing 10 SSCP-negative samples per exon; however, even if we calculated that 10% of p53 mutations were missed, the total incidence would rise to only 31%, which is still lower than the average incidence of p53 mutations in lung cancer (56%), as reported by Greenblatt et al (1994). Our results indicate that p53 mutations in NSCLC may be caused by carcinogens other than those found in tobacco smoke. This may account for the population-specific mutation spectrum.

Sixty-seven per cent of the NSCLC samples in this study demonstrated p53-positive staining. It is of note that all but one of the patients' tumours with p53 mutations, excluding those with insertions or deletions, also demonstrated p53-positive staining. This provides further evidence for the hypothesis that there is an increased *p53* gene product in tumours containing mutations in the *p53* gene. The p53-positive staining is considered to represent stable p53 protein overexpression that results from post-translational modification and/or p53 protein complexing. However, there were also NSCLC tumours that demonstrated p53-positive staining but in which no mutations were found. These differences

© Cancer Research Campaign 1997

between molecular and immunohistochemical results may be explained by the possibility that there are mutations in the *p53* gene outside exons 4–9, within the p53 promoter region, or that overexpression of p53 is not only due to *p53* mutations but also to some other factors, e.g. mdm2, which bind to the p53 protein and thus increase its half-life. Furthermore, it may be argued that the DO-1 antibody may also detect a stable conformationally altered wild-type p53 protein which, in its own right, may lead to genetic instability without an initial *p53* mutation.

More than half (6 of 11) of base substitutions occur at G residues, which is in agreement with previous reports (Greenblatt et al, 1994). However, in contrast with most previous reports, we found a higher prevalence of GC→AT transitions (37.5% of all mutations) than of GC→TA transversions (12.5%), which would be expected from a smoking population. Comparison of the ratio of GC→TA to GC→AT in this study and Greenblatt's review showed a significant difference ($P = 0.04$). Husgafvel-Pursiainen et al (1995) reviewed the mutational profile in smokers and ex-smokers among lung cancer patients and found that 34% of *p53* mutations were G→T base substitutions. In this study, however, only 18% of the smokers had G→T transversions. Findings similar to our own were reported in NSCLC patients from Taiwan, in whom G→T mutations constituted only 6% of *p53* mutations detected in a population sample comprising 61% smokers (Lee et al, 1994). GC→TA transversions have been attributed to the action of benzo[*a*]pyrene, a member of the polycyclic aromatic hydrocarbons, which represent the major carcinogens found in tobacco smoke. However, other carcinogens, such as 4-(methylnitrosamino)-1-(3-pyridyl)-1-butanone (NNK), cause GC→AT transitions and GC→TA transversions at CpG and non-CpG sites, depending on the experimental system used (Wynder and Hoffmann, 1994). Tobacco smoke is a complex mixture of compounds, and the mutational spectrum it causes needs further investigation (DeMarini et al, 1995). Hence, it remains unclear whether or not the prevalence of GC→AT transitions found in the smoking population in this study is a paradox. It is possible that, rather than one factor acting alone, a combination of one or many environmental components and genetic factors may act synergistically.

Strikingly, in this study all four C→T and one out of three G→A mutations (five out of seven GC→AT in total) occurred at non-CpG dinucleotides (in this case in CpC/GpG dinucleotides; Table 2). CpG sites can account for mutations due to spontaneous deamination of 5-methylcytosine residues, but the reason for the occurrence of GC→AT transitions at non-CpG sites is unclear. As little is known about methylation at non-CpG sites in mammalian cells (Tasheva and Roufa 1994), it is uncertain whether GC→AT transitions at non-CpG sites represent induced or spontaneous mutagenesis (Yang et al, 1996).

No correlation was found between the presence of mutations and the stage of tumour, indicating that p53 mutations may be considered to play a role in the early development of lung cancer. However, it may also be argued that cells containing *p53* mutations could be harbouring a mutation in another gene that may merely have a selective growth advantage in vivo, and this would result in the late amplification of a p53-mutant subset of cells in a mixed-status tumour cell population. The presence of only one 'non-smoker' in our samples did not allow us to undertake any comparison of p53-positive staining and a history of smoking vs non-smoking; however, a trend was found between p53 immunostaining and a history of heavy smoking (76–126 pack–years) compared with light smoking (0–45 pack–years), but this was found to be not statistically significant ($P = 0.13$).

*p53* mutations did not correlate with age, alcohol consumption or prognosis (median 18 months' follow-up). However, a trend was found between the prevalence of *p53* mutations and squamous carcinomas, which is in agreement with a previous report by Mitsudomi et al (1993). The absence of a correlation between *p53* mutations and various clinicopathological parameters indicates that a mutation in the *p53* gene may not by itself lead to aggressive cell growth. Recently, an inverse correlation between clinical outcome and p53-positive immunostaining was found; however, no correlation was found between *p53* mutations and survival (Carbone et al, 1994). It is also probable that these tumours carry additional alterations in genes that function as oncogenes or tumour-suppressor genes; indeed, the allelotype study for this set of tumours showed a median FAL value of 0.09, indicating allelic imbalance throughout the genome (Neville et al, 1996). In particular, LOH at the TP53 locus, as shown in this study, was found in 25% of these tumours. It is of note that only two out of ten samples with LOH at TP53 were found to contain a mutation in p53, implying that another candidate tumour-suppressor gene may also exist at this locus. Additionally, four mutations in codon 12 of the K-*ras* gene have also been demonstrated in these tumours (Neville et al, 1995*a*). Only one of the tumours was found to contain mutations in both the K-*ras* and the *p53* genes.

In conclusion, *p53* mutations in the population of the Merseyside region of the UK do not appear to be as common as in other reports of NSCLC. GC→AT transitions in p53, preferentially occurring at non-CpG sites, suggest that different environmental carcinogens may be involved in this geographic area.

### ACKNOWLEDGEMENTS

This work was supported by the Roy Castle Foundation, Liverpool, UK. The authors would like to thank Professor David Lane for generously providing the DO-1 antibody.

### REFERENCES

Carbone DP, Mitsudomi T, Chiba I, Piantadosi S, Rusch V, Nowak JA, McIntire D, Slamon D, Gazdar A and Minna J (1994) p53 immunostaining positivity is associated with reduced survival and is imperfectly correlated with gene mutations in resected non-small cell lung cancer. A preliminary report of LCSG 871. *Chest* **106**: 377S–381S

Chiba I, Takahashi T and Nau MM (1990) Mutations in the p53 gene are frequent in primary, resected non-small cell lung cancer. *Oncogene* **5**: 1603–1610

DeMarini DM (1983) Genotoxicity of tobacco-smoke and tobacco-smoke condensate. *Mutat Res* **114**: 59–89

DeMarini DM, Shelton ML and Levine LG (1995) Mutation spectrum of cigarette smoke condensate in Salmonella: comparison to mutations in smoking-associated tumours. *Carcinogenesis* **16**: 2535–2542

Field JK, Stocker CJ, Neville EN and Taylor LA (1994) Molecular mechanisms in lung cancer. In *International Congress for Lung Cancer*, Antypas G (ed.), pp. 29–34. Monduzzi Editor International Proceedings Division: Bologna

Field JK, Kiaris H, Risk JM, Tsiriyotis C, Adamson R, Zoumpourlis V, Rowley H, Taylor K, Whittaker J, Howard P, Beirne JC, Gosney JR, Woolger J, Vaughan ED, Spandidos DA and Jones AS (1995) Allelotype of squamous cell carcinoma of the head and neck: fractional allele loss correlates with survival. *Br J Cancer* **72**: 1180–1188

Field JK, Neville EM, Stewart MP, Swift A, Liloglou T, Risk JM, Ross H, Gosney JR and Donnelly RJ (1996) Fractional allele loss data indicate distinct genetic populations in the development of non-small cell lung cancer. *Br J Cancer* **74**: 1968–1974

Greenblatt MS, Bennett WP, Hollstein M and Harris CC (1994) Mutations in the p53 tumor suppressor gene: clues to cancer etiology and molecular pathogenesis. *Cancer Res* **54**: 4855–4878

© Cancer Research Campaign 1997 

Hollstein M, Sidransky D, Vogelstein B and Harris CC (1991) p53 mutations in human cancers. *Science* **253**: 49–53

Hollstein M, Rice K, Soussi T, Fuchs R, Sorlie T, Hivig E, Smithsorenson B, Montesano R and Harris CC (1994) Database of p53 gene somatic mutations in human tumours and cell lines. *Nucl Acids Res* **22**: 3551–3555

Husgafvel-Pursiainen K, Ridanpaa M, Anttila S and Vainio H (1995) p53 and ras gene mutations in lung cancer: implications for smoking and occupational exposures. *J Occup Environ Med* **37**: 69–76

Lane DP (1992) p53, guardian of the genome. *Nature* **358**: 15–16

Lee LN, Shew JY, Sheu JC, Lee YC, Lee WC, Fang MT, Chang HF, Yu CJ, Yang PC and Luh KT (1994) Exon-8 mutation of p53 gene associated with nodal metastasis in non-small cell lung cancer. *Am J Respir Crit Care Med* **150**: 1667–1671

Lehman TA, Bennett WP, Metcalf RA, Welsh JA, Ecker J, Modali RV, Ullrich S, Romano JW, Appella E, Testa JR, Gerwin BI and Harris CC (1991) p53 mutations, ras mutations, and p53-heat shock 70 protein complexes in human lung carcinoma cell lines. *Cancer Res* **51**: 4090–4096

Levine AJ, Momand J and Finlay CA (1991) The p53 tumour suppressor gene. *Nature* **351**: 453–456

Lohmann D, Putz B, Reich U, Bohm J, Prauer H and Hofler H (1993) Mutational spectrum of the p53 gene in human small-cell lung cancer and relationship to clinicopathological data. *Am J Pathol* **142**: 907–915

Mitsudomi T, Steinberg SM, Nau MM, Carbone D, Damico D, Bodner S, Oie HK, Linnoila RI, Mulshine JL, Minna JD and Gazdar AF (1992) p53 gene-mutations in non-small-cell-lung-cancer cell-lines and their correlation with the presence of ras mutations and clinical features. *Oncogene* **7**: 171–180

Mitsudomi T, Oyama T, Kusano T, Osaki T, Nakanishi R and Shirakusa T (1993) Mutations of the p53 gene as a predictor of poor prognosis in patients with non small cell lung cancer. *J Natl Cancer Inst* **85**: 2018–2023

Neville EM, Ellison G, Kiaris H, Stewart MP, Spandidos DA, Fox JC and Field JK (1995*a*) Detection of K-ras mutations in non-small cell lung carcinoma. *Int J Oncol* **7**: 511–514

Neville EM, Stewart MP, Myskow M, Donnelly RJ and Field JK (1995*b*) Loss of heterozygosity at 9p23 defines a novel locus in non-small cell lung cancer. *Oncogene* **11**: 581–585

Neville EM, Stewart MP, Swift A, Risk JM, Liloglou T, Ross H, Gosney JR, Donnelly RJ and Field JK (1996) Allelotype of non-small cell lung cancer. *Int J Oncol* **9**: 533–539

Ramet M, Castren K, Jarvinsen K, Pekkala K, Turpeenniemi-Hujanen T, Soini Y, Paakko P and Vahakangas K (1995) p53 protein expression correlated with benzoapyrene-DNA adducts in carcinoma cell lines. *Carcinogenesis* **16**: 2117–2124

Sameshima Y, Matsuno Y, Hirohashi S, Shimosato Y, Mizoguchi H, Sugimura T, Terada M and Yokota J (1992) Alterations of the p53 gene are common and critical events for the maintenance of malignant phenotypes in small-cell lung carcinoma. *Oncogene* **7**: 451–457

Shopland DR, Eyre HJ and Pechacek TF (1991) Smoking-attributable cancer mortality in 1991: is lung cancer the leading cause of death among smokers in the United States? *J Natl Cancer Inst* **83**: 1142–1148

Sidransky D and Hollstein M (1996) Clinical implications of the p53 gene. *Ann Rev Med* **47**: 285–301

Suzuki H, Takahashi T, Kuroishi T, Suyama M, Ariyoshi Y, Takahashi T and Ueda R (1992) p53 mutations in non-small cell lung cancer in Japan: association between mutations and smoking. *Cancer Res* **52**: 734–736

Takeshima Y, Seyama T, Bennett WP, Akiyama M, Tokuoka S, Inai K, Mabuchi K, Land CE and Harris CC (1993) p53 mutations in lung cancers from nonsmoking atomic bomb survivors. *Lancet* **342**: 1520–1521

Tasheva ES and Roufa DJ (1994) Densely methylated DNA islands in mammalian chromosomal replication origins. *Mol Cell Biol* **14**: 5636–5644

Westra ES, Offerhaus GJA, Goodman SN, Slebos RJC, Polak M, Baas IO, Rodenhuis S and Hruban RH (1993) Overexpression of the p53-tumour-supressor gene-product in primary lung adenocarcinomas is associated with cigarette smoking. *Am J Surg Pathol* **17**: 213–220

Whitehouse JMA (1994) Management of Lung Cancer Current Clinical Practices 1994, Vol. 1, pp. 1–58. Department of Health: London

Williams EMI, Youngson J, Ashby D and Donelly RJ (1992) Lung cancer bulletin – A framework for action. Merseyside and Cheshire Cancer Registry: Liverpool

Wynder EL and Hoffmann D (1994) Smoking and lung cancer: scientific challenges and opportunities. *Cancer Res* **54**: 5284–5295

Yang AS, Gonzalgo ML, Zingg J-M, Millar RP, Buckley JD and Jones PA (1996) The rate of CpG mutations in Alu repetitive elements within the p53 tumour suppressor gene in the primate germline. *J Mol Biol* **258**: 240–250

Yin YX, Tainsky MA, Bischoff FZ and Strong LC (1992) Wild-type p53 restores cell-cycle control and inhibits gene amplification in cells with mutant p53 alleles. *Cell* **70**: 937–948

Youngson J and Williams EMI (1995) Incidence of lung cancer in Merseyside and Cheshire (1989–1993). Merseyside and Cheshire Cancer Registry: Liverpool

Zheng J, Shu QP, Li ZH, Tsao JL, Weiss LM and Shibata D (1994) Patterns of p53 mutations in squamous-cell carcinoma of the lung – acquisition at a relatively early age. *Am J Pathol* **145**: 1444–1449

© Cancer Research Campaign 1997